# Kinetics of slow release of nitrogen fertiliser from multi-layered nanofibrous structures

**DOI:** 10.1038/s41598-021-84460-x

**Published:** 2021-03-01

**Authors:** Leila Javazmi, Anthony Young, Gavin J. Ash, Tobias Low

**Affiliations:** 1 School of Mechanical and Mechatronic Engineering, University of Southern Queensland, Toowoomba, Australia; 2 Centre for Crop Health, University of Southern Queensland, Toowoomba, Australia; 3 School of Agriculture and Food Sciences, The University of Queensland, Saint Lucia, Australia

**Keywords:** Structural properties, Two-dimensional materials

## Abstract

Fertilisers are essential in modern agriculture to enhance plant growth, crop production and product quality. Recent research has focused on the development of delivery systems designed to prolong fertiliser release. This study introduces a new technology to encapsulate and release molecules of fertilisers by using multi-layered electrospun nanofibre as a carrier. Single-layer poly L-lactic acid (PLLA) nanofibres loaded with urea were fabricated using electrospinning. Triple-layer nanofibrous structures were produced by electrospinning polyhydroxybutyrate (PHB) nanofibres as external layers with PLLA nanofibres impregnated with urea fertiliser as the middle layer. Scanning electron microscopy (SEM) and Fourier transform infrared spectrophotometry (FTIR) were employed to characterize the morphology of electrospun nanofibres. Urea release dynamic was analysed using a total nitrogen instrument (TNM-1). The results indicated that triple-layered urea-impregnated nanofibrous structures led to lower initial rate of nitrogen release and slower release rate of cumulative nitrogen which extended for more than three months. It is concluded that triple-layer nanofibrous structures have the potential for slow release delivery of fertilisers.

## Introduction

Nanofibres are filaments with diameters in the nanometre range and an aspect ratio (length: diameter) larger than 100:1^1,2^. This intrinsic features of nanofibres increases the surface area to volume ratio and decreases spaces between individual nanofibres compared to regular fibres^3,4^. There are several methods used to prepare nanofibres, encompassing both top-down (melt-blown, melt electrospinning, islands-in-the-sea, and electrospinning) and bottom-up (interfacial polymerization, self-assembly, and phase separation) approaches^5^.

Electrospinning is a production technique to prepare ultrafine fibres at a micro- or nanoscale. It has the ability to spin a broad range of polymers, the potential for upscale, and provides precise control over morphology, aspect ratio, pore-size distribution and porosity in comparison to other approaches to forming continuous nanofibres^6,7^. Furthermore, electrospun nanofibres are ideal porous membranes given their open-pore structure (tens of nanometres to one micrometre), high surface area and high gas permeability. Such nanofibres have been used in areas as diverse as filtrations, nanocomposites, drug delivery, biomedical, medical prostheses, fuel cells, sensors, and protective clothing^5,8^. However, the use of electrospun nanofibers in agriculture is novel and still in its infancy^9^.

Fertilisers are used to promote plant growth, increase crop production and improve quality of products. The officious use of nitrogenous fertiliser is hindered by system losses occurring through volatilization and leaching^10^. Recent investigations have focused on the development of systems using different materials that prolong the release of fertilisers^11^. These systems aim to minimise soil contamination by controlling fertiliser release using low cost sustainable materials^10^. Electrospun nanofibres have a very high specific surface area which makes them promising candidates for delivery of agrichemicals^12–14^. Electrospun nanofibres have an advantage over particulate carriers to control fertiliser encapsulation as they are less likely to be washed away than nanoparticles^9,15^. As a result, agricultural producers can potentially decrease the amount of fertiliser loss and prevent potential environmental contamination, as well as fertiliser run-off, by using nanofibrous networks^15^.

Urea is a cost-effective solid nitrogen-based fertiliser used to promote plant growth and increase crop production. However, uncontrolled excessive use of urea fertiliser is harmful to plants and can lead to soil and water pollution^12^. Thus, significant research efforts have focused on prolonged-released systems that minimise adverse environmental impacts and increase the efficiency of urea fertiliser use^16^.

Electrospun nanofibres have been demonstrated as potential vehicles for agrichemical delivery. Urea impregnated onto wheat gluten electrospun-fibre membranes was found to have a rapid release in the first 10 min, followed by a decreased rate until equilibrium at 5 h, when 98% was released^16^. While demonstrating the potential for nanofibre delivery of agrichemicals, the release dynamics were not suitable for slow-release fertilisers^17^. An advancement was coaxial electrospinning where urea was incorporated in the PLLA core surrounded by a PHB sheath. This released urea for a month and biodegraded within three months^18^. To the best of our knowledge, no research has reported encapsulation and release of agricultural chemicals from triple-layered nanofibrous structures. The focus of this study was to provide proof of concept of this novel approach to the encapsulation of urea within a triple-layered nanofibrous matrix.

## Results and discussion

### Electrospinning of single-layered and triple-layered nanofibre mats loaded with urea

A custom electrospinning apparatus was used to produce single-layered PLLA nanofibres loaded with different concentrations of urea. The PLLA solution was fixed at 5% (w/w), while urea was loaded at 10%, 20%, and 40% (w/w), relative to the mass amount of PLLA, and transferred to a 1 mL syringe with an attached 18-gauge blunt tip needle. Single-layered PLLA nanofibres loaded with different urea concentrations were collected onto an aluminium foil surface and stored in a desiccator under vacuum for 24 h prior to use.

Triple-layer nanofibre mats consisting of a layer of PLLA sandwiched between PHB layers were prepared. A PHB polymeric solution with concentration of 7% (w/w) was fed into the electrospinning apparatus using a 1 mL syringe to produce the first PHB nanofibre layer on the surface of aluminium foil collector. Subsequently, 5% PLLA solution with 5%, 10%, 20% and 40% (w/w) urea was electrospun onto the PHB nanofibre layer. Finally, the outer surface of PLLA nanofibre layer was electrospun by another layer of PHB nanofibre resulting in a PHB/PLLA/PHB triple-layer nanofibrous structure.

### Nanofibre characterization

SEM (FEI Quanta 200 SEM 2002) at the Royal Melbourne Institute of Technology (RMIT) Microscopy and Microanalysis Facility (RMMF) and benchtop SEM (JEOL JCM-6000PLUS) at the University of Southern Queensland (USQ) were used to determine the morphology of electrospun nanofibres. Image J processing software was used to measure PLLA and PHB nanofibre diameters from high magnification SEM images.

The morphology of electrospun nanofibres is dependent on electrospinning parameters including applied voltage, solution composition, solution concentration, solution feed rate and collection distance^19^. When the concentration of a polymeric solution increases, the solution viscosity increases^20^. By keeping all electrospinning parameters constant, and increasing the concentration of a polymeric solution, nanofibres are formed thicker as the higher viscosity solution which prevents the polymer jet from stretching further and electrospinning thinner fibres^21^.

**Figure 1:**
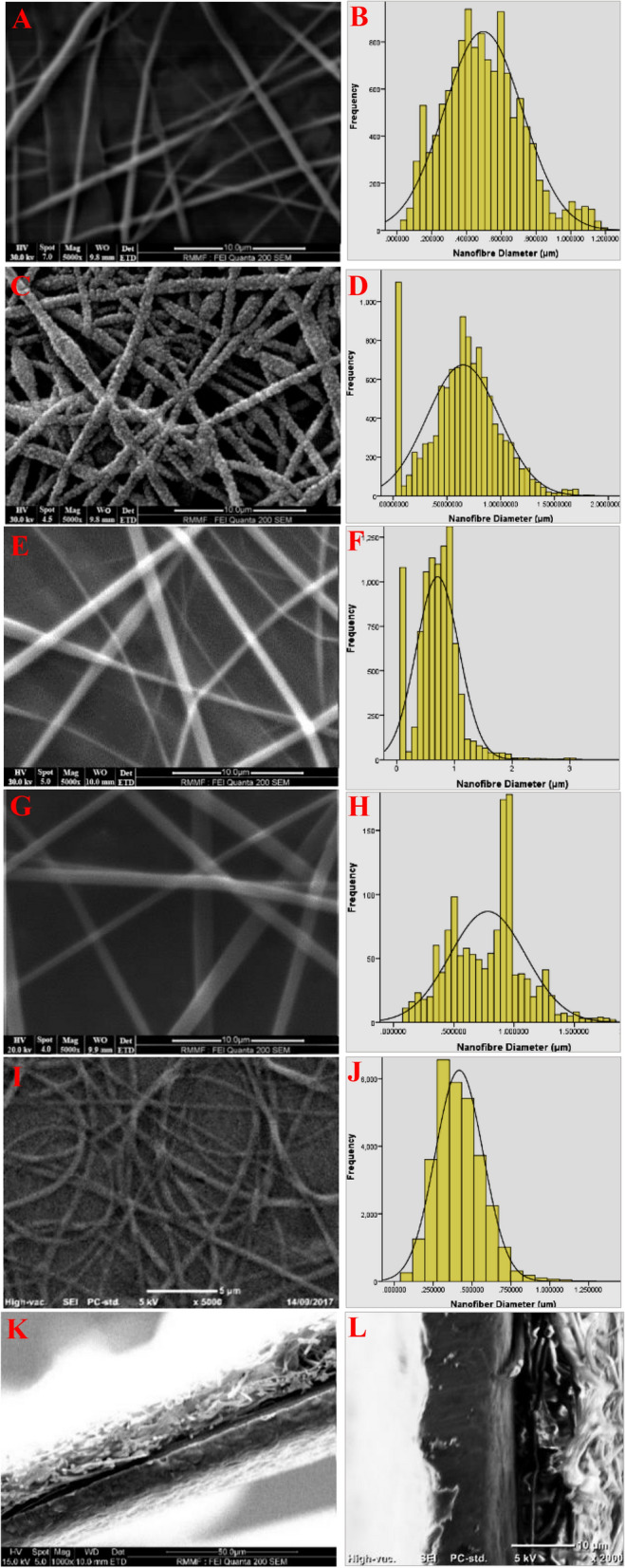
SEM images and related diameter histograms of PLLA electrospun nanofibres containing urea at concentrations of 0% *(**A**,**B**), 10% **(**C**,**D**), 20% *(**E**,**F**), and 40% *(**G**,**H**). SEM images and diameter distribution of 7% PHB electrospun nanofibres (**I**,**J**). SEM image of cross section of PHB/PLLA/PHB triple-layer nanofibrous structure containing 10% urea at (**K**) 1000 × magnification, and (**L**) 2000 × magnification. * Gold coating** Carbon coating.

In this study, the effect of urea concentration on nanofibre morphology was investigated. The diameter of PLLA electrospun nanofibres increased from 496 to 782 nm as urea concentration increased from 0 to 40% (Fig. 1, Table 1). The morphology and diameter distribution of 7% PHB electrospun nanofibres are shown in Fig. 1I,J. The average diameters of the outer layer PHB nanofibres were 418 ± 64 nm. A typical SEM micrograph cross section of triple-layer PHB/PLLA/PHB containing 10% urea is illustrated in Fig. 1K,L. The thickness of the triple-layer nanofibrous structure is approximately 21 μm. The micrograph illustrates the nanofibre arrangement and void spaces in the triple-layered structure.

**Table 1:** Effect of urea concentration on electrospun nanofibre diameter.

Urea concentration (*% w/w)	Nanofibre diameter (nm)	**CV%
0	496.183	22%
10	650.154	33%
20	710.000	37%
40	782.231	31%

*% w/w based on amount of PLLA used.

**Coefficient of Variation (CV).

Brunauer–Emmett–Teller (BET) analysis of the 5% PLLA electrospun nanofibres without loading urea indicated that the porous fibrous mat was fabricated with a surface area of 188 m^2^/g, a mean pore diameter of 37 nm and a total pore area of 10 m^2^/g. Thus, the structure satisfies the definition for nanofibrous structures^22^.

**Figure 2:**
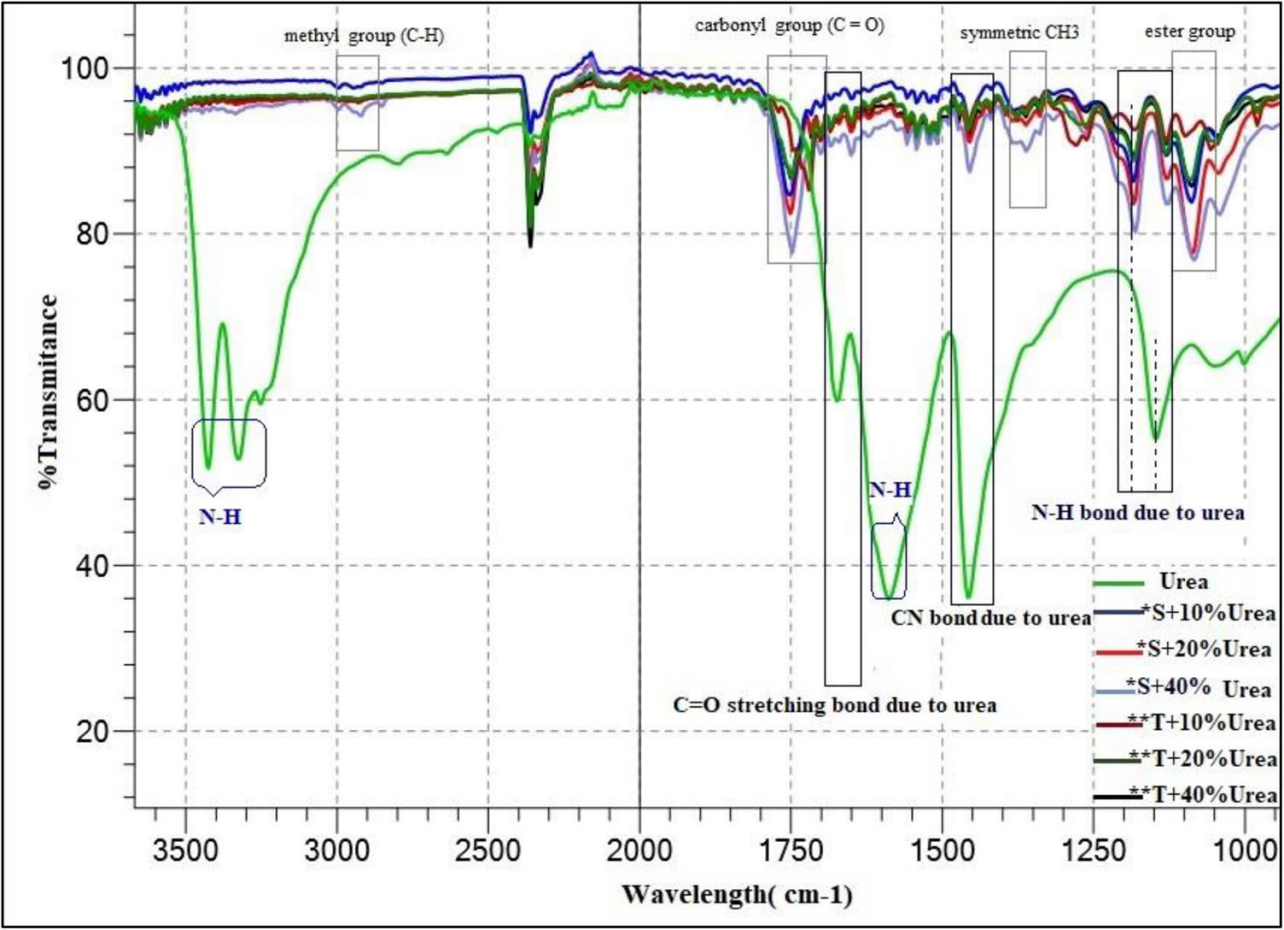
FTIR spectra of urea and single-, triple-layered PLLA nanofibres loaded with urea. Pure urea (Urea), PLLA nanofibre containing 10% urea (*S+ 10%Urea), PLLA nanofibre containing 20% urea (*S+ 20%Urea), PLLA nanofibre containing 40% urea (*S+ 40%Urea), PHB/PLLA/PHB nanofibre containing 10% urea (**T+ 10%Urea), PHB/PLLA/PHB nanofibre containing 20% urea (**T+ 20%Urea), PHB/PLLA/PHB nanofibre containing 40% urea (**T+ 40%Urea). *S: Single-layered PLLA nanofibre mat, **T: Triple-layered PHB/PLLA/PHB nanofibrous structure.

The FTIR spectrograph of urea powder and single- and triple-layered PLLA nanofibres loaded with 10%, 20% and 40% urea is shown in Fig. 2. Characteristic peaks of PLLA form at 1090–1190 cm^−1^ (ester bond), a middle peak at 1362 cm^−1^ (–CH3 symmetric bending vibration), and two peaks at 2997 and 2947 cm^−1^ (methyl groups)^23^. A characteristic strong peak of PLLA at about 1750 cm^−1^ is related to the stretching vibration of the carbonyl group (C=O) shifted slightly from 1755 cm^−1^^24^. In the FTIR spectra of urea, the peak in the region of 1629–1680 cm^−1^ is due to the C=O stretching bond of urea and the presence of absorption peaks at 3344, 3447 and 1157 cm^−1^ can be associated to the stretching vibration of N–H bonds^25^. The characteristic peaks of urea at 1458 cm^−1^ and approximately 1596 cm^−1^ reflect the stretching vibration of the C–N bond and N–H bonding, respectively^26^. The urea characteristic peaks at 1458 cm^−1^ (C–N bond), 1157 cm^−1^ (N–H bond) and between 1629–1680 cm^−1^ (C=O stretching) are all present in the FTIR spectrums of single- and triple-layered nanofibrous structures. However, peak positions of C=O and N–H bonds in PLLA nanofibres have been shifted due to changes in vibrational frequency which can occur through changes in bond strength or in reduced mass of urea^27^.

### Urea release characteristics

**Figure 3:**
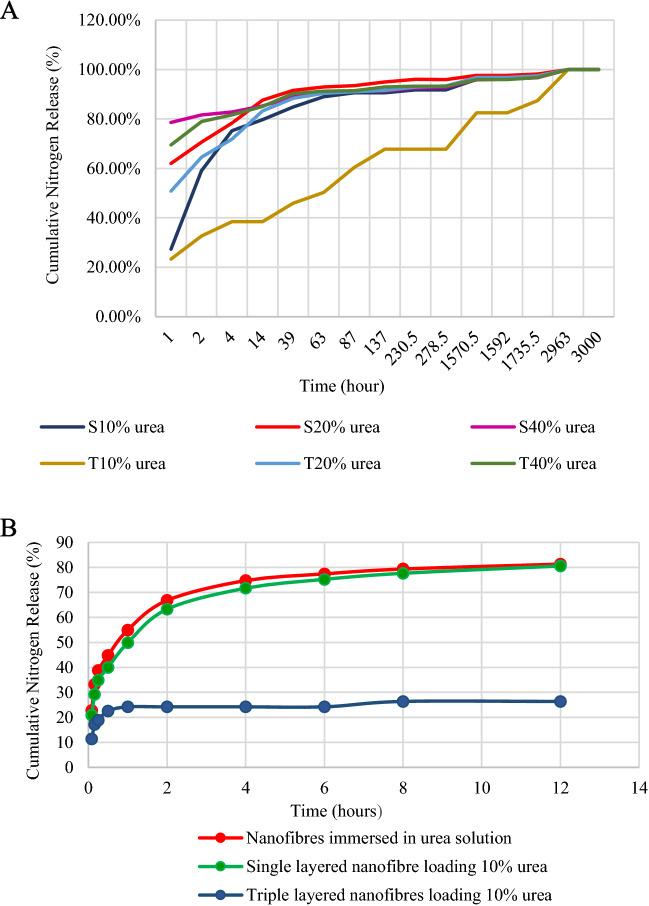
(**A**) Cumulative nitrogen release from single- and triple-layer nanofibre structures from first hour until 3000 hours, and (**B**) Cumulative nitrogen release from immersed nanofibres in urea solution and single-, triple-layer nanofibrous structure containing 10% urea from first hour until 12 hours. *S* single-layered PLLA nanofibre mat, *T* triple-layered PHB/PLLA/PHB nanofibrous structure.

A TNM-1 total nitrogen instrument (Shimadzu) was used to determine the cumulative percentage of nitrogen released from single- and triple-layer nanofibres containing 10%, 20%, and 40% urea, respectively (Table 2). Increasing urea percentage from 10% to 40% in both single-layered PLLA nanofibre mat, and triple-layered PHB/PLLA/PHB nanofibrous structures, resulted in initial nitrogen release at a higher rate (*P* value = 0.000) (Table 2). The nitrogen release rate of single-layered nanofibres was not significantly affected by various urea concentration (*P* value  = 0.361) (Fig. 3A), however, increasing urea concentration into triple-layered nanofibres resulted in significantly higher nitrogen release rate (*P* value  = 0.000). Statistical analysis of the results (using a one-way ANOVA) for cumulative nitrogen release showed that at different urea concentrations, triple-layered nanofibres containing 10% urea showed a highly significant decrease in release rate compared with other samples (*P* value  = 0.000).

**Table 2:** Nitrogen release rate from single- and triple-layer nanofibre structures from first hour till 3000 h.

Time (h)/sample	S10% urea	S20% urea	S40% urea	T10% urea	T20% urea	T40% urea
1	27.30%	62.00%	78.60%	23.30%	50.80%	69.50%
2	59.10%	70.70%	81.60%	32.70%	64.60%	79.00%
4	75.20%	78.30%	82.80%	38.50%	71.80%	81.70%
14	79.70%	87.60%	85.30%	38.50%	83.20%	85.10%
39	84.80%	91.60%	88.90%	45.90%	88.40%	90.30%
63	89.00%	93.00%	91.00%	50.30%	90.40%	91.30%
87	90.60%	93.50%	91.20%	60.40%	91.00%	91.50%
137	90.60%	95.00%	91.40%	67.80%	91.60%	93.00%
230.5	91.80%	96.00%	92.50%	67.80%	93.00%	93.30%
278.5	91.80%	95.90%	92.80%	67.80%	93.40%	93.30%
1570.5	96.10%	97.60%	95.80%	82.50%	96.80%	95.90%
1592	96.10%	97.60%	96.20%	82.50%	96.80%	95.90%
1735.5	97.20%	98.20%	96.70%	87.40%	97.40%	96.80%
2963	100.00%	100.00%	100.00%	100.00%	100.00%	100.00%
3000	100.00%	100.00%	100.00%	100.00%	100.00%	100.00%
*P* value for initial nitrogen release	0.000			0.000		

*S* single-layered PLLA nanofibre mat, *T* triple-layered PHB/PLLA/PHB nanofibrous structure, *P* value probability value.

For PLLA impregnated with 10% w/w urea, after 39 hours the triple-layered nanofibre mats had released less than 50%, while the single layer mats had released over 80%. Based on these results, the experiment was repeated for single- and triple-layered nanofibrous structure containing 10% urea. Figure 3B shows cumulative nitrogen release from single- and triple-layered nanofibres loaded with 10% urea as well as the control sample for the first 12 hours. Results revealed that triple-layer nanofibrous structure containing 10% urea released nitrogen at a slower rate compared to single-layer nanofibrous structure encapsulating urea by either electrospinning fabrication or immersing deposition. The physical barrier of PHB nanofibre layers coating PLLA nanofibre layer resulted in slower rate of urea release in triple-layered nanofibre containing 10% urea compared to single-layered PLLA nanofibre. Furthermore, increasing the urea content into single- and triple-layered PLLA nanofibres resulted in increasing bead numbers in samples^28^. Thus, fabricated multi-layered nanofibres can be considered a means of delaying urea release from a PLLA nanofibrous matrix.

## Conclusion

Single- and triple-layer nanofibrous structure were fabricated and loaded with urea using a custom-built electrospinning device. Fourier transform infrared Spectrophotometer analysis showed that urea was encapsulated in all samples. As expected, increased urea concentration led to larger nanofibre diameters. Increasing the percentage of urea loaded into nanofibres from 10% to 40% increased the initial rate of nitrogen release. Both single- and triple- layered nanofibres samples released nitrogen for three months. Although high urea concentrations did not affect the nitrogen release rate for single-layer nanofibres significantly (*P* value  = 0.361), increasing urea content into triple-layered nanofibres resulted in increasing nitrogen release content significantly (*P* value  = 0.000). Triple-layer nanofibrous structures containing 10% urea exhibited a significantly lower level and lower initial rate of nitrogen release compared to single-layer nanofibres loading 10% urea (*P* value  = 0.012). In conclusion, triple-layered electrospun PLLA nanofibres containing urea may be an effective carrier to control the release of urea fertiliser in agriculture applications. These materials have the potential as slow-release delivery systems for high-value agrochemicals.

## Methods

### Materials

Poly (L-lactide) (PLLA) with a molecular weight of 282kD was purchased from Vorina Biomaterials Company in Ireland (CAS Number: 33135-50-1). Poly [(R)-3-hydroxybutyric acid] (PHB) was provided from Sigma Aldrich (Product Number: 363502). Solvents, N-Dimethylformamide (DMF); Reagent Plus®, ≥ 99%, chloroform (CF); anhydrous, ≥ 99%. Acetone (AC) for HPLC, ≥ 99.8% were obtained from Australia Sigma Aldrich. Urea (N: P: K; 46-0-0) was purchased from Richgro Garden Products. The amount of nitrogen is equal to 46% urea mass.

### Preparation of PLLA and PHB solutions

PLLA solution with optimum concentrations of 5% (w/w) in chloroform: acetone (3:1 v/v) was prepared and mixed with 10, 20, and 40% (w/w) urea powder relative to the weight of PLLA. Polyhydroxybutyrate polymeric solution in dimethylformamide: chloroform (30:70 v/v) solvents was prepared at concentration of 7% (w/w).

### Electrospinning apparatus

**Figure 4:**
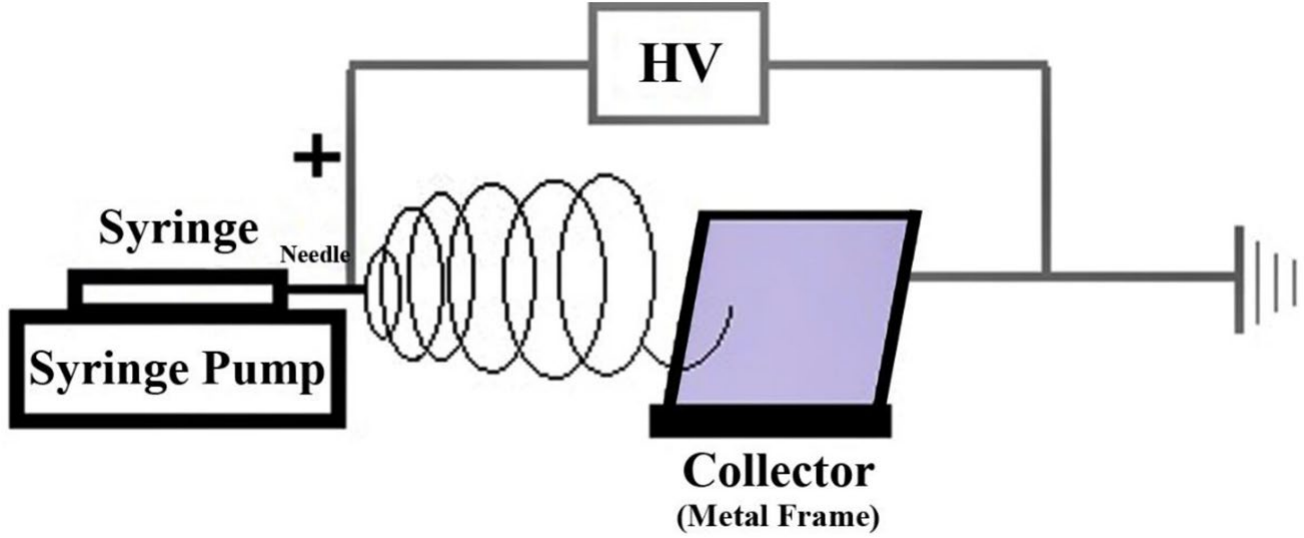
Schematic diagram of the electrospinning apparatus.

The schematic setup for nanofibre electrospinning is shown in Fig. 4. It consists of a high voltage power supply, model 73,030, DC input 30 kV @ 1 mA, (Genvolt, Ireland), and a New Era NE-300 “Just Infusion” syringe pump. A metal frame 14 cm × 16 cm with attached aluminium foil is located 15 cm from the syringe needle to collect the nanofibres. The positive terminal of the power supply is connected to the needle and the ground terminal is attached to the collector (metal frame). The electrospinning process occurs between the needle tip and the aluminium collector and nanofibres gather on the surface of the aluminium foil.

Electrospinning was conducted at 32 ºC with 12 (+ kV) high voltage, and 20 (+ kV) high voltage for PLLA and PHB solutions, respectively. Feed rate and needle size for both solutions were selected at 1 mL/hr and 18 G. The morphology of electrospun nanofibres was determined by varying the urea concentration ranging from 0% to 40% in a fixed 5% PLLA solution.

### Characterisation

Accelerated Surface Area and Porosimetry System (ASPA) 2400 Micropore Data Reduction was used to study BET analyses for the 5% PLLA electrospun nanofibre to evaluate surface areas, pore diameters and total pore area.

FTIR measurements of urea powder and single-, triple-layered PLLA nanofibres loaded with 10%, 20% and 40% urea were carried out using IRAffinity-1S Fourier transform infrared Spectrophotometer (Shimadzu). Aluminium foil was used to place the samples under spectrophotometer.

The single- and triple-layered nanofibrous mats with a thickness of approximately 7 and 21 μm, respectively, were cut into 2 cm × 6 cm pieces. These pieces were accurately weighed ± 0.00001 g and placed in a plastic tube with 20 mL of milli-Q water to soak for 2 minutes to wash out any deposited urea fertiliser on the surface of the nanofibrous mats. Subsequently, each sample was immersed in a 50 mL plastic tube with 20 mL milli-Q water and placed in a shaker at 70 RPM and 30°C^18^. The samples were removed from the shaker and immersed in the next tube containing 20 mL milli-Q water at different periods, ranging from 0 up to 3000 hours. A TNM-1 total nitrogen instrument (Shimadzu) was used to measure nitrogen release from the nanofibre mats. The data were reported as percent nitrogen. Cumulative nitrogen release from each sample was calculated for the determined period. A 5% PLLA nanofibre mat without loading urea immersed in 0.5% urea solution for 24 hours, was used as the control sample. All experiments were carried out three times and results were reported as average ± one standard deviation.
